# Complete genome sequence of *Solobacterium moorei* 10714-02 isolated from the stool of a human colorectal cancer patient

**DOI:** 10.1128/mra.00981-25

**Published:** 2026-01-20

**Authors:** Ayana Shinomiya, Kota Oshibuchi, Jiayue Yang, Shinji Fukuda, Kazuharu Arakawa

**Affiliations:** 1 Institute for Advanced Biosciences, Keio University, Tsuruoka, Yamagata, Japan; 2 Faculty of Environment and Information Studies, Keio University 12869, Fujisawa, Kanagawa, Japan; 3 Gut Environmental Design Group, Kanagawa Institute of Industrial Science and Technology 34762, Kawasaki, Kanagawa, Japan; 4 Systems Biology Program, Graduate School of Media and Governance, Keio University 12869, Fujisawa, Kanagawa, Japan; 5 MIRAI Technology Institute, Shiseido Co., Ltd., Yokohama, Japan; 6 Transborder Medical Research Center, Institute of Medicine, University of Tsukuba, Tsukuba, Ibaraki, Japan; 7 Innovative Microbiome Therapy Research Center, Juntendo University Graduate School of Medicine, Bunkyo-ku, Tokyo, Japan; Loyola University Chicago, Chicago, Illinois, USA

**Keywords:** *Solobacterium moorei*, complete genome, nanopore sequencing, colorectal cancer

## Abstract

We report the complete circular genome sequence (2,316,546 bp; 37.0% G + C) of *Solobacterium moorei* 10714-02, a gram-positive obligate anaerobe isolated from stool samples of a human colorectal cancer patient, differing from the typically linear chromosome of this species.

## ANNOUNCEMENT

*Solobacterium moorei* is a gram-positive, anaerobic oral commensal bacterium that is associated with sepsis, bacteremia, halitosis, and periodontal disease (1, 2). *S. moorei* may migrate to the colon, where it could contribute to the progression of colorectal cancer (3). Here, we report the complete genome sequence of *S. moorei* 10714-02, isolated from fecal samples of colorectal cancer patients.

Isolation was performed as previously described (4), using a selective medium prepared as follows. The selective medium consisted of 59 g/L Gifu Anaerobic Medium (GAM) broth, 15 g/L agar, 0.1 g/L rifampicin, 0.05 g/L gentamicin, 0.1 g/L kanamycin, and 0.1 g/L neomycin. Colonies were screened with PCR targeting the *S. moorei*-specific region of the 16S rRNA using the primers SomF (5′-TCGGAAGGCATCTTCTGGTT-3′) and SomR (5′-AAGTGGCTGGATTGGGTTGA-3′) (5). *S. moorei* 10714-02 was in GAM at 37°C in an anaerobic environment for 24 h, confirmed by 16S rRNA sequencing using the universal bacterial primers 27F (5′-AGAGTTTGATCMTGGCTCAG-3′) and 1492R (5′-TACGGYTACCTTGTTACGACTT-3′) (4), and stored as a glycerol stock. The samples used in this study were obtained under conditions of informed consent and with approval of the institutional review boards of each participating institute (National Cancer Center, 2013-244; Tokyo Institute of Technology, 2014018). The genomic DNA was extracted using the NucleoBond high-molecular-weight DNA kit (Macherey-Nagel) according to the manufacturer’s protocol. The sequencing library was prepared using the Rapid Barcoding Sequencing Kit (SQK-RBK114; Oxford Nanopore Technologies) and sequenced using an R10.4.1 Flowcell (FLO-MIN114) on a MinION Mk1B device with MinKNOW v.24.11.8 (Oxford Nanopore Technologies). Basecalling and demultiplexing were performed using Dorado v.0.9.0 in Super Accurate Mode. Adapter sequences were trimmed using Porechop v.0.2.4 (6), resulting in 1.3 Gbp consisting of 494,318 reads with an N50 length of 16,630 bp. Reads longer than 50,000 bp filtered with reformat.sh tool in BBMap (v38.84) (7), which had 54.8× coverage, were used for assembly using Canu v.2.3 (8). The genome was assembled into a single contig and circularized as specified by the Canu output, but 29 raw reads also supported the circularity. The draft assembly was error corrected in one round using all sequenced reads with Medaka v1.11.3 (Oxford Nanopore Technologies) (9). The completeness of the assembly was evaluated with CheckM v.1.2.2 (10) that resulted in 100% completeness (k__Bacteria [UID2372]), and genome was rotated so that the *dnaA* gene comes first and was annotated using the DDBJ Fast Annotation and Submission Tool (DFAST) server (11). All software was used with default settings unless specified otherwise.

The assembled genome had a length of 2,316,546 bp with a G + C content of 37.0%, containing 2,275 CDSs, 9 rRNAs, and 43 tRNAs. Strain 10714-02 was confirmed to be *S. moorei*, where average nucleotide identity was calculated to be 97.47% with *S. moorei* DSM22971 (GCA_000425005.1) using the DFAST server (10). *S. moorei* genomes reported so far have linear chromosomes, but this strain possesses a circular chromosome.

## Data Availability

The genome sequence was deposited in DDBJ under accession number AP043952, and the raw reads were deposited in the Sequence Read Archive (SRA) under BioProject accession number PRJNA1300821 as SRR34846837 run. https://www.ncbi.nlm.nih.gov/bioproject/?term=PRJNA1300821 https://getentry.ddbj.nig.ac.jp/getentry/na/AP043952/.

## References

[1] Kageyama A, Benno Y. 2000. Phylogenic and phenotypic characterization of some eubacterium-like isolates from human feces: description of Solobacterium moorei gen. nov., sp. nov. Microbiol Immunol 44:223–227. doi:10.1111/j.1348-0421.2000.tb02487.x 10832964

[2] Vancauwenberghe F, Dadamio J, Laleman I, Van Tornout M, Teughels W, Coucke W, Quirynen M. 2013. The role of Solobacterium moorei in oral malodour. J Breath Res 7:046006. doi:10.1088/1752-7155/7/4/046006 24185504

[3] Uchino Y, Goto Y, Konishi Y, Tanabe K, Toda H, Wada M, Kita Y, Beppu M, Mori S, Hijioka H, Otsuka T, Natsugoe S, Hara E, Sugiura T. 2021. Colorectal cancer patients have four specific bacterial species in oral and gut microbiota in common-a metagenomic comparison with healthy subjects. Cancers (Basel) 13:3332. doi:10.3390/cancers13133332 34283063 PMC8268706

[4] Haraszthy VI, Gerber D, Clark B, Moses P, Parker C, Sreenivasan PK, Zambon JJ. 2008. Characterization and prevalence of Solobacterium moorei associated with oral halitosis. J Breath Res 2:017002. doi:10.1088/1752-7155/2/1/017002 21386146

[5] Yoshihide F, Fuchigami M, Tsuzukibashi O. 2020. Isolation and identification methods for Solobacterium moorei involved in halitosis. J Jpn Soc Evid Dent Prof 12:11–21. doi:10.15041/jsedp.12.11

[6] Wick RR, Judd LM, Gorrie CL, Holt KE. 2017. Completing bacterial genome assemblies with multiplex MinION sequencing. Microb Genom 3:e000132. doi:10.1099/mgen.0.000132 29177090 PMC5695209

[7] Bushnell B. 2025. BBMap. Available from: https://sourceforge.net/projects/bbmap. Retrieved 28 Mar 2025.

[8] Koren S, Walenz BP, Berlin K, Miller JR, Bergman NH, Phillippy AM. 2017. Canu: scalable and accurate long-read assembly via adaptive k-mer weighting and repeat separation. Genome Res 27:722–736. doi:10.1101/gr.215087.116 28298431 PMC5411767

[9] Stanojević D, Lin D, Nurk S, Sessions P, Šikić M. 2024. Telomere-to-telomere phased genome assembly using HERRO-corrected simplex nanopore reads. bioRxiv. doi:10.1101/2024.05.18.594796:2024.05.18.594796 PMC13323052 42045451

[10] Parks DH, Imelfort M, Skennerton CT, Hugenholtz P, Tyson GW. 2015. CheckM: assessing the quality of microbial genomes recovered from isolates, single cells, and metagenomes. Genome Res 25:1043–1055. doi:10.1101/gr.186072.114 25977477 PMC4484387

[11] Tanizawa Y, Fujisawa T, Nakamura Y. 2018. DFAST: a flexible prokaryotic genome annotation pipeline for faster genome publication. Bioinformatics 34:1037–1039. doi:10.1093/bioinformatics/btx713 29106469 PMC5860143

